# Research priorities for liver glycogen storage disease: An international priority setting partnership with the James Lind Alliance

**DOI:** 10.1002/jimd.12178

**Published:** 2019-11-13

**Authors:** Fabian Peeks, Willemijn F. Boonstra, Lut de Baere, Camilla Carøe, Thomas Casswall, Damián Cohen, Katherine Cowan, Iris Ferrecchia, Alberto Ferriani, Caroline Gimbert, Marcus Landgren, Nerea L. Maldonado, Jason McMillan, Antal Nemeth, Niccolò Seidita, Ute Stachelhaus‐Theimer, David A. Weinstein, Terry G. J. Derks

**Affiliations:** ^1^ Section of Metabolic Diseases, Beatrix Children's Hospital University Medical Center Groningen, University of Groningen Groningen The Netherlands; ^2^ Belgische Organisatie voor Kinderen en volwassenen met een Stofwisselingsziekte VZW Belgium; ^3^ Paediatric Nutrition, University Hospital of Copenhagen Rigshospitalet Copenhagen Denmark; ^4^ Department of Pediatric Gastroenterology Hepatology and Nutrition, Karolinska University Hospital; ^5^ CLINTEC, Karolinska Institutet Stockholm Sweden; ^6^ Glucolatino, Rosario Argentina; ^7^ James Lind Alliance University of Southampton Southampton UK; ^8^ Glycogen Storage Disease Program University of Connecticut Farmington Connecticut; ^9^ Connecticut Children's Medical Center Hartford Connecticut; ^10^ Association for Glycogen Storage Disease; ^11^ Associação Brasileira de Glicogenose (ABGLICO) Brazil; ^12^ L'Association Francophone des Glycogénoses France; ^13^ Scandinavian Association for Glycogen Storage Disease (SAGSD) Sweden; ^14^ Asociación Española de Enfermos de Glucogenosis, General Practitioner Institut Català de la Salut (ICS) Barcelona Spain; ^15^ Association for Glycogen Storage Disease UK; ^16^ Associazione Italiana Glicogenosi Italy; ^17^ Selbsthilfegruppe Glykogenose Deutschland e.V. Germany

**Keywords:** caregivers, James Lind Alliance, liver glycogen storage diseases, patient participation, priority setting partnership, rare diseases, research, research priorities

## Abstract

The international liver glycogen storage disease (GSD) priority setting partnership (IGSDPSP) was established to identify the top research priorities in this area. The multiphase methodology followed the principles of the James Lind Alliance (JLA) guidebook. An international scoping survey in seven languages was distributed to patients, carers, and healthcare professionals to gather uncertainties, which were consolidated into summary questions. The existing literature was reviewed to ensure that the summary questions had not yet been answered. A second survey asked responders to prioritize these summary questions. A final shortlist of 22 questions was discussed during an international multi‐stakeholder workshop, and a consensus was reached on the top 11 priorities using an adapted nominal group technique.In the first survey, a total of 1388 questions were identified from 763 responders from 58 countries. These original uncertainties were refined into 72 summary questions for a second prioritization survey. In total 562 responders from 58 countries answered the second survey. From the second survey, the top 10 for patients, carers and healthcare professionals was identified and this shortlist of 22 questions was taken to the final workshop. During the final workshop, participants identified the worldwide top 11 research priorities for liver GSD. In addition, a top three research priorities per liver GSD subtype was identified.This unique priority setting partnership is the first international, multilingual priority setting partnership focusing on ultra‐rare diseases. This process provides a valuable resource for researchers and funding agencies to foster interdisciplinary and transnational research projects with a clear benefit for patients.

**Abbreviations**
DM: diabetes mellitus
GSD: glycogen storage disease
IGSDPSP: international GSD priority setting partnership
JLA: James Lind Alliance
PSP: priority setting partnership

## 1 INTRODUCTION

Liver glycogen storage diseases (GSD) are ultra‐rare diseases, among the oldest known inborn errors of metabolism described in literature, and classified according to the protein deficiency and the organ distribution.^1^ Liver GSD subtypes include GSD 0, Ia, Ib, III, IV, VI, IX, and XI, and classical clinical presentations of patients include severe fasting intolerance, growth failure, and hepatomegaly. Biochemically, liver GSD is associated with hypoglycemia, hyperlactatemia, increased liver enzymes, and hyperlipidemia. Long‐term complications include liver adenomas, nephropathy, cardiomyopathy, and severe muscle symptoms. Strict dietary management is the cornerstone of management to maintain normal blood glucose concentrations, to suppress secondary metabolic derangements and to prevent long‐term complications.^2^ Although we now understand details of the diseases that we did not some decades ago, we are still missing important information in many areas of the field.

The international GSD community has a longstanding tradition of involving patient representatives in directing healthcare and research.^3^, ^4^ However, there are discrepancies between questions considered relevant by patients, carers, and healthcare professionals, and the research performed in rare diseases.^5^ The James Lind Alliance (JLA) was set up in 2004 to facilitate partnerships between patients, carers, and healthcare professionals and help to identify research priorities. The JLA developed a process for identifying these research uncertainties that are important to either patients, carers, and/or clinicians. In a “priority setting partnership (PSP),” the JLA methodology works equally with these stakeholders to prioritize research uncertainties to guide future funding and investments.^6^ The JLA methodology has been used in almost 100 other areas of healthcare and has shown differences in research priorities between on one side researchers and on the other patients, carers, and healthcare professionals (http://www.jla.nihr.ac.uk/priority-setting-partnerships/).

To address research priorities of direct relevance and potential benefit to liver GSD patients, carers, and the treating healthcare providers, the international liver glycogen storage disease priority setting partnership (IGSDPSP) was initiated on 11 November 2016. The IGSDPSP has been the first international, multilingual PSP to identify and prioritize uncertainties in a group of ultra‐rare diseases. Here, we report the process and outcomes of this partnership including the top 11 research priorities in the field of liver GSD, agreed upon by patients, carers, and clinicians.

## 2 METHODS

The Medical Ethical Committee of the University Medical Centre Groningen confirmed that the Law on Medical Scientific Research involving Human Beings (WMO) did not apply to the IGSDPSP (METc 2017/386).

**Figure 1:**
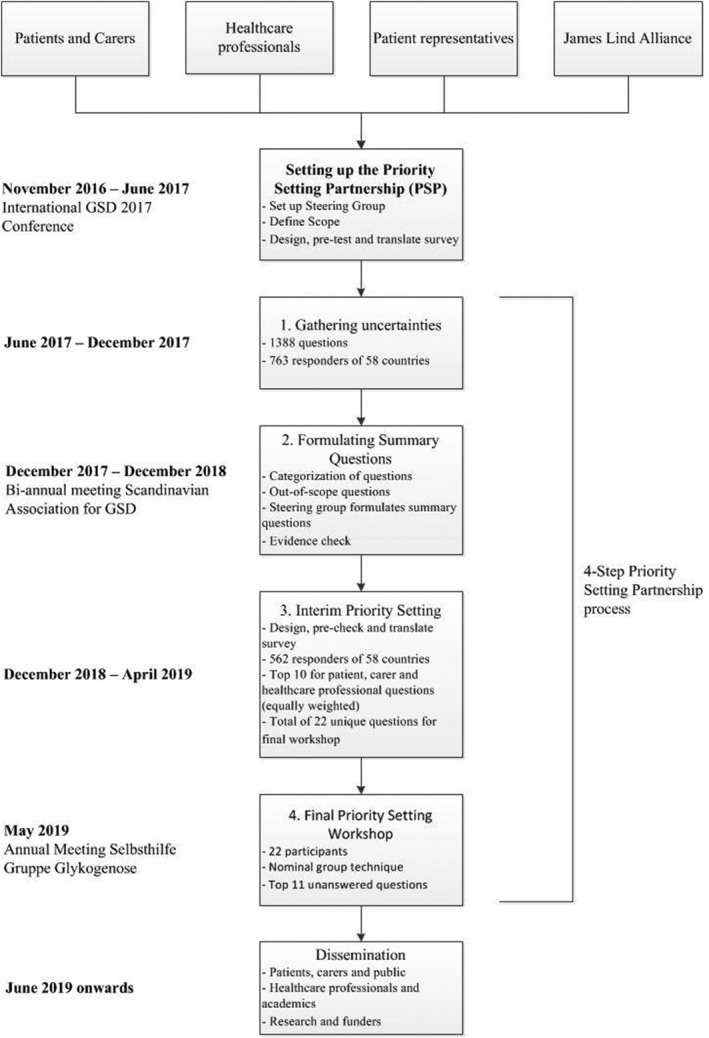
Flow chart of the international liver GSD priority setting partnership process

For 30 months, the multiphase methodology followed the principles of the JLA guidebook,^7^ as depicted in Figure 1 and on our website (http://igsdpsp.com). Prior to the start of the PSP, key challenges were determined in the readiness questionnaire (File S1). After the senior author (T.D.) reaching out to the JLA, potential partners such as patients, individuals that care for patients (carers), patients/carers active in national patient organizations (patient representatives), and healthcare professionals (physicians, dieticians, nurses) were contacted and informed of the establishment and aims of the IGSDPSP. People who showed interest were invited to attend and participate in the initial awareness meeting and the first steering group meeting during the International GSD conference in Groningen (the Netherlands) held on 15 to 17 June 2017. Before the initial awareness meeting, this open invitation was repeated during an oral presentation by the JLA advisor (K.C.) at one of the plenary sessions. The steering group had consisted of 14 members from 12 countries of whom some represented more than one stakeholder: 10 carers or patients, 9 healthcare professionals (7 physicians, 1 nurse, 1 dietician), and 8 representatives of patient organizations. The group was chaired and facilitated by K.C. and two information specialists (F.P., W.F.B.), who analysed and categorized the raw data from the surveys. In total, 11 telephone conferences and 3 in‐person meetings took place. Phases of the process were closely monitored and guided by the steering group. The JLA Priority Setting Partnership four‐step process was followed, involving (a) gathering uncertainties, (b) formulating summary questions, (c) interim priority setting, and (d) final priority setting (Figure 1).

### 2.1 Gathering uncertainties

An online international scoping survey to gather uncertainties was designed via SurveyMonkey (a digital platform for designing and distributing surveys) and underwent pretesting and refinement with the steering group and selected members of the liver GSD community (File S2). Afterwards, the survey was translated into seven languages (Dutch, English, French, German, Italian, Portuguese, and Spanish) by native speakers from the steering group. From 1 October to 14 December 2017, this first survey was distributed to patients, carers, and healthcare professionals by multiple partner organizations, such as patient organizations, professional networks, and individual patients, carers, and healthcare professionals. The survey requested up to three answers to the question: “What are your questions on the care and/or management of liver Glycogen Storage Disease?” Furthermore, basic demographic data were collected (age of patient[s], role, type of liver GSD, country of origin/work). The collected uncertainties were categorized qualitatively based on the UK Clinical Research Collaboration Health Classification System (diagnosis, prevention, prognosis, education, health services, social care, self‐management, and treatment) and further subcategorized according to the specific topic. Questions considered to be “out‐of‐scope” were removed at this stage after agreement by the steering group.

### 2.2 Formulating summary questions

The categorized uncertainties were collected into a small number of summary questions during the second in‐person steering group meeting at the Scandinavian bi‐annual patient meeting for patients with liver GSD (SAGSD) in Ängelholm (Sweden) on 28 and 29 April 2018. The process was overseen by JLA Advisor (K.C.), emphasizing that the data should be treated with neutrality and transparency. An extensive literature search was performed to ensure that these summary questions had not been answered. The search focused on information from available reviews, guidelines, and a PubMed search strategy containing Medical Subject Heading (MESH) terms from each summary question.

### 2.3 Interim priority setting

A second online prioritization survey was designed via SurveyMonkey, refined with the steering group and translated into seven languages (File S3). From 19 March to 27 April 2019, the prioritization survey was launched and asked respondents to prioritize the summary questions. The distribution was performed via the same partner organizations used for the first survey and through participants from the first survey who sent their contact details. Responders were asked in a two‐step process to choose their top 10 research questions on the care and/or management of liver GSD. Again, basic demographic data were collected (age of patient[s], role, type of liver GSD, country of origin/work). Questions were ranked ordinally and if two questions ranked equally, the joint rank was given. The top 10 questions for patients, carers, and healthcare professionals were selected separately for the final prioritization workshop to include priorities for each group, accounted for discrepancies between groups and illustrated the importance of shared‐decision taking at the final workshop.

### 2.4 Final priority setting

Via an open call on the IGSDPSP website, social media, and by steering group members (including D.A.W. and T.D.), people with liver GSD, carers, and healthcare professionals were suggested as participants for the final priority setting workshop at the Selbsthilfegruppe Glykogenose Deutschland e.V. in Duderstadt (Germany) on 24 May 2019. The selection and invitation was overseen by the independent JLA advisor (K.C.). Participants who initiate research, and thus already had the possibility to influence the research agenda, were excluded from participation in the final workshop, at which D.A.W. and T.D. were only observers and did not participate. Furthermore, all participants in the final workshop (Table 1) were fully reimbursed for their travel and accommodation costs to reduce economical and geographical bias. Participants were selected to represent the international liver GSD community and the GSD subtypes. Participants declared that they did not have any conflict of interest that might influence the priority setting process. The workshop was guided by three trained JLA advisors (including K.C.) using an adapted nominal group technique.^7^, ^8^ Participants were divided in three smaller groups comprising of different stakeholders and ranked questions in two rounds. Between rounds, the average ranking of the questions were determined by F.P. and K.C., after which the questions were presented in the ranked order in the second round. In a final plenary session, the participants were able to comment on the ranking and were able to revise the order one final time.

**Table 1:** Demographic details on responders of the first and second prioritization survey

	First survey		Second survey		Final workshop	
Responders or participants (#)	Total	763^a^	Total	562	Total	22
	Patients	150	Patients	86	Patients	5
	Carers	370	Carers	253	Carers	12
	HCP	266	HCP	166	HCP	11
	Do not want to share	15	Do not want to share	4		
	Other	11	Other	0		
	No answer	0	No answer	53		
The healthcare professionals identified as	Physicians	139	Physicians	105	Physicians	4
	Nurses	5	Nurses	11	Nurses	2
	Dieticians	80	Dieticians	41	Dieticians	4
	Do not want to share	5	Do not want to share	2	Psychologist	1
	Other	4	Other	7		
	No answer	33	No answer	0		
GSD subtype the patient or carer represented (#)	GSD 0	4	GSD 0	8	GSD 0	1
	GSD Ia	212	GSD Ia	124	GSD Ia	8
	GSD Ib	71	GSD Ib	50	GSD Ib	2
	GSD III	65	GSD III	61	GSD III	2
	GSD IV	4	GSD IV	5	GSD IV	0
	GSD VI	9	GSD VI	10	GSD VI	1
	GSD IX	68	GSD IX	45	GSD IX	3
	GSD XI	1	GSD XI	2	GSD XI	0
	Unclassified/unknown:	23	Unclassified/unknown:	11	Unclassified/unknown:	0
	Do not want to share	3	Do not want to share	1		
	Other	11	Other	5		
	No answer	39	No answer	17		
Age of patient in years (median; min‐max)	12; 0‐75		12; 0‐64			
HCP years of experience (#)	0‐5	73	0‐5	51		
	6‐10	40	6‐10	31		
	11‐15	43	11‐15	31		
	16‐20	20	16‐20	9		
	>20	83	>20	35		
	Do not want to share	5	Do not want to share	5		
	No answer	38	No answer	4		
Country^b^	Total: 58 Afghanistan = 1; Argentina = 21; Australia = 4; Austria = 6; Belgium = 3; Brazil = 58; Canada = 37; Chile = 8; China = 1; Colombia = 11; Croatia = 1; Czech Republic = 2; Denmark = 11; Ecuador = 6; Egypt = 1; Faroe Islands = 5; France = 15; Georgia = 3; Germany = 75; Greece = 3; Guatemala = 1; India = 2; Iraq = 3; Israel = 4; Italy = 12; Lithuania = 1; Mexico = 21; Nepal = 3; Netherlands = 32; Nicaragua = 2; Norway = 2; Oman = 1; Pakistan = 1; Peru = 4; Philippines = 2; Poland = 1; Portugal = 6; Romania = 1; Russian Federation = 1; Saudi Arabia = 1; Singapore = 3; Slovakia = 1; Slovenia = 3; South Africa = 1; South Sudan = 4; Spain = 34; Swaziland = 5; Sweden = 28; Switzerland = 2; Togo = 2; Tunisia = 2; Turkey = 3; United Arab Emirates = 1; United Kingdom of Great Britain and Northern Ireland = 11; United Republic of Tanzania = 8; United States of America = 190; Uruguay = 1.		Total: 58 Afghanistan = 1; Angola = 1; Argentina = 12; Australia = 6; Austria = 7; Bangladesh = 1; Belgium = 5; Bolivia = 1; Brazil = 58; Canada = 11; Chile = 6; China = 1; Colombia = 8; Costa Rica = 1; Croatia = 1; Czech Republic = 3; Denmark = 7; Dominican Republic = 2; Ecuador = 8; Egypt = 1; Estonia = 2; Finland = 1; France = 20; Germany = 45; Greece = 4; Grenada = 1; Guatemala = 1; India = 1; Ireland = 3; Italy = 10; Malaysia = 1; Mexico = 23; Netherlands = 21; Norway = 1; Oman = 1; Pakistan = 1; Peru = 4; Philippines = 1; Poland = 1; Portugal = 3; Saudi Arabia = 1; Slovenia =1; South Africa = 6; Spain = 31; Sweden = 7; Switzerland = 6; Syrian Arab Republic = 2; Thailand = 1; Tunisia = 2; Turkey = 6; United Kingdom of Great Britain and Northern Ireland = 34; United States of America = 117; Uruguay = 1; Venezuela = 2; Yemen = 1.		Total: 11Argentina = 1; Belgium = 1; Brazil = 1; Denmark = 1; Germany = 4; Mexico = 1; the Netherlands = 5; Spain = 1; Sweden = 1; UK = 1; USA = 5.	

Abbreviations: #, total number of responders; HCP, healthcare professional.

a Responders to the survey were able to select more than one role (for example for families responding to the survey together).

b Total number of countries including the number of responders per country.

## 3 RESULTS

Table 1 presents the demographics for the responders from both surveys and the final workshop participants.

### 3.1 Gathering uncertainties

In the first survey, a total of 1388 questions were identified from 763 responders from 58 countries.

### 3.2 Formulating summary questions

The questions identified through the first survey were categorized by the information specialists (F.P. and W.F.B.). Afterwards, each individual question was defined as in‐scope or out‐of‐scope. Of the 1388 questions, 505 were deemed out‐of‐scope by the information specialists and the steering group (File S4). During the SAGSD, the steering group reconvened and formulated 72 summary questions from the remaining individual in‐scope questions in three groups (File S5 and S6). Of these out‐of‐scope questions, the steering group made sure that the topics raised were covered in the summary questions. Each group included at least a patient or carer, healthcare professional and a patient representative. Afterwards, the evidence check ascertained that the summary questions were unanswered.

### 3.3 Interim priority setting

Of these 72 summary questions, the top 10 for each stakeholder group (patients, carers, and healthcare professionals) were identified and taken to the final priority setting workshop, resulting in a shortlist of 22 summary questions (File S6).

### 3.4 Final priority setting

The final workshop participants agreed upon the top 11 priorities for liver GSD together via open and respectful discussions (Table 2). First, the participants agreed on the importance of including questions on subtypes of liver GSD and voted unanimously that an 11th question should be added on the prevention of muscle problems—an important topic for GSD IIIa. Second, the steering group and final workshop participants decided to present the top 3 research priorities for each subtype, in addition to the general top 11 research priorities. The top three research priorities for GSD subtypes was based on the results from the second survey to represent the ultra‐rare subtypes of liver GSD (ie, GSD 0, IV, VI, XI) (Table 3). If there were multiple questions that had a joint rank in the top 3, we prioritized the questions that were highest in the overall ranking after the second survey. Third, the final workshop participants emphasized that the research question on quality of life should not be a single priority, but they acknowledged quality of life as an overarching priority in liver GSD.

**Table 2:** Top 11 priorities for research in liver GSD, in rank order of priority

		Listed rank after the second survey		
Rank	Priority	Patients	Carers	HCP
1	What are the best options (eg, gene therapy or enzyme replacement therapy) for achieving sufficient amount of working enzyme in patients with liver GSD?	3J	6J	12J
2	Can consensus guidelines (for management) be achieved for patients with liver GSD?	71	58J	8J
3	How should optimal metabolic control both clinically and biochemically (like lactate, ketones, and/or lipids) be achieved in liver GSD?	20J	32J	5J
4	How should sickness and emergency situations be managed for patients with liver GSD?	9J	7	18
5	What is the best way to start dietary treatment, finding the optimal doses, and to administer the diet for patients with liver GSD?	34J	37J	10J
6	How can existing cornstarch preparations be modified or alternative treatments be implemented that are easier to administer and/or keep blood sugar levels more stable for patients with liver GSD?	9J	4	4
7	What is the role for new methods for monitoring metabolic control (like noninvasive continuous glucose and lactate measurements, new biomarkers) for patients with liver GSD?	40J	24J	8J
8	How to manage diet regimen in relation to "before, during and after" physical exercise (sport, playing) for patients with liver GSD?	5J	3	14
9	What are the long‐term complications (liver, renal, gut) of a diet rich in uncooked cornstarch and/or high protein and should the diet be adjusted to prevent complications in liver GSD?	9J	1	3
10	What are the risks and benefits of different options for overnight treatment for patients with liver GSD and how can we maximize safety?	48J	22J	10J
11	How to prevent and/or treat muscle problems in patients with liver GSD?	2	24J	22J

Abbreviations: HCP, healthcare professional; J, joint rank.

**Table 3:** Top three priorities for research in liver GSD subtypes

GSD type	Subtype rank	Rank after Q2^a^	Priority
GSD 0	1	23J	What are the consequences of consumption of alcohol and drugs for patients with liver Glycogen Storage Disease?
	2	29J	What (laboratory) testing and with which frequency is optimal for monitoring patients with liver Glycogen Storage Disease?
	3	5	How to manage diet regimen in relation to “before, during and after” physical exercise (sport, playing) for patients with liver Glycogen Storage Disease?
GSD Ia	1	2	What are the risks and benefits of gene therapy for patients with liver Glycogen Storage Disease?
	2	1	What are the long‐term complications (liver, renal, gut) of a diet rich in uncooked cornstarch and/or high protein and should the diet be adjusted to prevent complications in liver Glycogen Storage Disease?
	3	4	What are the best options (eg, gene therapy or enzyme replacement therapy) for achieving sufficient amount of working enzyme in patients with liver Glycogen Storage Disease?
GSD Ib	1	41	What is the best therapy for neutropenia and infections (ie, G‐CSF or alternatives considering outcomes), complications and side effects (ie, bone pain) in patients with Glycogen Storage Disease Type Ib (or Ia)?
	2	50J	What is the optimal therapy (Modulen or alternatives) for inflammatory bowel disease (IBD) and acute flares in patients with Glycogen Storage Disease Type Ib?
	3	16	How to better prevent and/or treat intestinal problems in patients with liver Glycogen Storage Disease?
GSD III	1	10	How to prevent and/or treat muscle problems in patients with liver Glycogen Storage Disease?
	2	20	What are the effects of different kinds of Ketogenic Diet in patients with Glycogen Storage Disease Type III?
	3	2	What are the risks and benefits of gene therapy for patients with liver Glycogen Storage Disease?
GSD IV	1	11	How is the (natural) progression of liver Glycogen Storage Disease at different stages of life?
	2	23J	When should liver transplantation be considered in patients with liver Glycogen Storage Disease and what are the (dis)advantages and long‐term outcomes?
	3	6J	What is the needed restriction of lactose, fructose or saccharose in different types of liver Glycogen Storage Disease?
GSD VI	1	43	How do body changes throughout life impact blood sugars in patients with liver Glycogen Storage Disease?
	2	23J	When should liver transplantation be considered in patients with liver Glycogen Storage Disease and what are the (dis)advantages and long‐term outcomes?
	3	50J	How can we personalize treatment for patients with liver Glycogen Storage Disease?
GSD IX	1	1	What are the long‐term complications (liver, renal, gut) of a diet rich in uncooked cornstarch and/or high protein and should the diet be adjusted to prevent complications in liver Glycogen Storage Disease?
	2	6J	What is the needed restriction of lactose, fructose or saccharose in different types of liver Glycogen Storage Disease?
	3	5	How to manage diet regimen in relation to “before, during and after” physical exercise (sport, playing) for patients with liver Glycogen Storage Disease?
GSD XI	1	39	How can all healthcare providers involved (including experts) contribute to shared care for individual patients with liver Glycogen Storage Disease?
	2	3	How can existing cornstarch preparations be modified or alternative treatments be implemented that are easier to administer and/or keep blood sugar levels more stable for patients with liver Glycogen Storage Disease?
	3	1	What are the long‐term complications (liver, renal, gut) of a diet rich in uncooked cornstarch and/or high protein and should the diet be adjusted to prevent complications in liver Glycogen Storage Disease?
Unclassified/unknown	1	3	How can existing cornstarch preparations be modified or alternative treatments be implemented that are easier to administer and/or keep blood sugar levels more stable for patients with liver Glycogen Storage Disease?
	2	5	How to manage diet regimen in relation to “before, during and after” physical exercise (sport, playing) for patients with liver Glycogen Storage Disease?
	3	10	How to prevent and/or treat muscle problems in patients with liver Glycogen Storage Disease?

Abbreviation: J, joint rank.

a Rank after the second prioritization survey, but before the final prioritization workshop.

## 4 DISCUSSION

We describe here the first international, multilingual PSP focusing on a group of ultra‐rare diseases. By involving patients, carers, and healthcare professionals from 73 countries, we have identified the top 11 research priorities for liver GSD (Table 2). In addition, priorities have been formulated for subtypes of liver GSD (Table 3). The majority of the top research priorities are relevant healthcare topics for many other inborn errors of metabolism and rare diseases in general. Our approach conveys a message towards researchers to invent new therapies and obtain worldwide management consensus by the different stakeholders, for example, patients, carers, and healthcare professionals.

With our approach—the multiphase PSP methodology following the JLA principles—patients, carers, and healthcare professionals jointly agreed on the top 11 research priorities. It is important to note that these priorities did not match those deemed by the professionals alone. Healthcare professionals prioritized, amongst other topics, metabolic control, new methods of metabolic monitoring and the role of dietary medium‐chain triglycerides. Patients and carers emphasized the importance of natural progression of disease and complications. Additionally, we were able to determine the top three research priorities per subtype of liver GSD (Table 3). Research priorities for GSD Ia focus on the gene therapy trial, whereas for GSD Ib and GSD III, on prevention of complications. Top priorities for the ketotic GSD subtypes 0, VI, and IX concern dietary restrictions and personalised treatment, and for the rarest GSD subtypes, that is, IV and XI, natural progression and treatment.

In 2012 and 2018 respectively, PSPs for diabetes mellitus (DM) type I^9^ and type II^10^ led to the identification of top 10 priorities. Given the existing similarities (in terms of monitoring glucose homeostasis, organization of health care, long‐term outcome) and differences (rarity, funding opportunities for research and reimbursement of basic health care for individual patients) between DM and liver GSD, it is interesting to compare the outcomes of the PSPs for these disorders. Similar to the top priority identified for DM type II, the top priority for liver GSD focuses on curing or reversing the condition. Furthermore, for DM Type I, the top priority is about the accurate monitoring of blood glucose concentrations, which is also mentioned twice in the top 11 (rank 3 and 7) research priorities for liver GSD. The similarities between the research priorities provide opportunities for shared research and healthcare projects that transcend the boundaries of one single disease.

Key challenges and limitations of our study were foreseen in the readiness questionnaire formulated at the start of the PSP (File S1). First, whereas in other PSPs, steering group members are from one country (ie, United Kingdom), our steering group members are from 12 countries, highlighting language differences both among IGSDPSP steering group members (50% were non‐native English speakers) and survey responders. Second, the surveys on liver GSD were self‐reported and therefore may overrepresent more severely affected patients. Third, although the JLA methodology strives to be transparent, open, and methodologically defensible, the approach has remained pragmatical and built upon the responses of end users. The qualitative analysis of the prioritization process is influenced by differences in response rates between individuals, countries, and cultures (Table 1). To reduce potential bias as much as possible, we widely distributed the surveys via professional and patient networks including social media, not limiting them to GSD patient organization members. Furthermore, both the steering group and the final workshop participants originated from multiple countries from Europe, North America, and South America. Patients, carers, and healthcare professionals have been consulted extensively throughout the entire process and the priorities are a result from a consensus decision taking process.

Since several stakeholders, including patients, carers, patient representatives, healthcare professionals, researchers, and funders, will be involved in answering the top priorities, we think it is important to inform them on the results of the PSP. To assure this, the steering group discussed the strategy for dissemination to reach these stakeholders after the final workshop. The strategy included this detailed scientific manuscript, a lay report, a social media and website campaign, press‐releases via patient organizations and channels from the JLA, and planned activities at the International GSD Conference in Porto Alegre, Brazil, from 14 to 16 November 2019. The steering group will continue to serve as a platform to disseminate the results from our PSP to a broader audience and to both monitor and share information on future research projects that result from these top priorities. In the future, funding for these research priorities should be addressed by stakeholders in national and international grant applications for basic, translational, clinical, and public health research projects.

Currently, global funding in the biomedical research field involves billions of dollars and millions of people.^11^ In this landscape, eminent scientists who obtain funding determine the course of research and this has not changed in the past decades.^12^, ^13^ Ideally decisions about research funding should take patients, carers, and healthcare professionals into consideration,^11^ but often they are not involved in the choice, design, performance, analysis, and dissemination of research.

Since liver GSD is a group of inborn errors of metabolism with extremely low prevalence, we consider our IGSDPSP as a proof of principle for ensuring stakeholder participation, and patient empowerment in rare and ultra‐rare diseases, in particular within the metabolic community. In these areas, more than in others, healthcare and research need to be intimately connected with all stakeholders.^14^ The IGSDPSP shows the importance to formulate research priorities together with patients, carers and healthcare professionals to share each other's point of view. We believe that our approach is essential for defining research priorities and for advancing research and treatment in rare diseases.

## CONFLICT OF INTEREST

None.

## AUTHOR CONTRIBUTIONS

Being members of the Steering Group, Lut de Baere, Camilla Carøe, Thomas Casswall, Damián Cohen, Iris Ferrecchia, Alberto Ferriani, Caroline Gimbert, Marcus Landgren, Nerea López Maldonado, Jason McMillan, Antal Nemeth, Niccolò Seidita, Ute Stachelhaus‐Theimer and David Weinstein, substantially contributed to the work, and were involved in (a) conception and design of the priority setting partnership, the surveys, and analysis and interpretation of data, and (b) revising the article critically for important intellectual content. All authors approved the final manuscript as submitted and agree to be accountable for all aspects of the work. All authors confirm the absence of previous similar or simultaneous publications. In addition, F.P. and W.F.B. were information specialists, K.C. was the JLA Advisor and T.G.J.D. was the PSP Lead in this partnership. F.P. coordinated this project, collected, and analyzed the data, wrote the first version of the manuscript, drafted, and wrote the final version of the manuscript. T.G.J.D. initiated this project, wrote the first version of the manuscript, drafted, and critically reviewed the later versions of the manuscript.

## ETHICS STATEMENT

The Medical Ethical Committee of the University Medical Centre Groningen confirmed that the Law on Medical Scientific Research involving human beings (WMO) did not apply to the International Liver Glycogen Storage Disease Priority Setting Partnership (METc 2017/386).

## Supporting information


**File S1.** IGSDPSP readiness questionnaire

**File S2.** First IGSDPSP survey for identification of uncertainties

**File S3.** Second IGSDPSP prioritization survey

**File S4.** Out‐of‐scope questions

**File S5.** Original questions of summary question “How should sickness and emergency situations be managed for patients with liver Glycogen Storage Disease?”
**File S6.** Total and group ranking of summary questions after the second prioritization survey. The top 10 and bottom 10 priorities are highlighted in green and red, respectively. *Rank after the second prioritization survey. HCP, Healthcare professional; J, joint rank

## References

[1] Weinstein DA , Steuerwald U , De Souza CFM , Derks TGJ . Inborn errors of metabolism with hypoglycemia: glycogen storage diseases and inherited disorders of gluconeogenesis. Pediatr Clin North Am. 2018;65(2):247‐265. 10.1016/j.pcl.2017.11.005. 29502912

[2] Walter J , Labrune PA , Laforet P . The glycogen storage diseases and related disorders In: Saudubray J, Baumgartner MR, Walter J, eds. Inborn Metabolic Diseases: Diagnosis and Treatment. Berlin, Heidelberg: Springer Berlin Heidelberg; 2016:121‐137. 10.1007/978-3-662-49771-5_5.

[3] Derks T , Nemeth AL , Adrian K , et al. Hepatic glycogen storage diseases: toward one global collaborative network. J Inborn Errors Metab Screen. 2017;5:1‐4. 10.1177/2326409817733009.

[4] Phillips A . More questions: 10 years later from glycogen storage disease patient support groups in Europe. Eur J Pediatr. 2002;161(1):S105 10.1007/BF02680005. 12373582

[5] Crowe S , Fenton M , Hall M , Cowan K , Chalmers I . Patients', clinicians' and the research communities' priorities for treatment research: there is an important mismatch. Res Involv Engagem. 2015;1(1):2 10.1186/s40900-015-0003-x. 29062491 PMC5598091

[6] Chalmers I , Atkinson P , Fenton M , Firkinds L , Crowe S , Cowan K . Tackling treatment uncertainties together: the evolution of the james lind initiative, 2003‐2013. J R Soc Med. 2013;106(12):482‐491. 10.1177/0141076813493063. 23824330 PMC3842854

[7] Cowan, K. , & Oliver, S. (2018). James Lind Alliance (JLA) Guidebook (Version 8 ed.). Southampton, England: University of Southampton.

[8] Gallagher M , Hares T , Spencer J , Bradshaw C , Webb I . The nominal group technique: a research tool for general practice? Fam Pract. 1993;10(1):76‐81. 10.1093/fampra/10.1.76. 8477899

[9] Gadsby R , Snow R , Daly AC , et al. Setting research priorities for type 1 diabetes. Diabet Med. 2012;29(10):1321‐1326. 10.1111/j.1464-5491.2012.03755.x. 22823450

[10] Finer S , Robb P , Cowan K , Daly A , Shah K , Farmer A . Setting the top 10 research priorities to improve the health of people with type 2 diabetes: a diabetes UK‐James Lind Alliance priority setting partnership. Diabet Med. 2018;35(7):862‐870. 10.1111/dme.13613. 29485717 PMC6032840

[11] Macleod MR , Michie S , Roberts I , et al. Biomedical research: increasing value, reducing waste. Lancet. 2014;383(9912):101‐104. 10.1016/S0140-6736(13)62329-6. 24411643

[12] Bol, T. , de Vaan, M. , & van de Rijt, A. (2018). The matthew effect in science funding. Proc Natl Acad Sci USA, 115(19), 4887‐4890. doi:10.1073/pnas.1719557115 29686094 PMC5948972

[13] Merton RK . The matthew effect in science. the reward and communication systems of science are considered. Science. 1968;159(3810):56‐63. 5634379

[14] Augustine EF , Dorsey ER , Saltonstall PL . The care continuum: an evolving model for care and research in rare diseases. Pediatrics. 2017;140(3):0108. 10.1542/peds.2017-0108 28818836

