# Supplementary material for: Research priorities for liver glycogen storage disease: An international priority setting partnership with the James Lind Alliance
Source: J Inherit Metab Dis. 2019 Nov 13;43(2):279–89. doi: 10.1002/jimd.12178 (PMC7079148; doi:10.1002/jimd.12178)
Supplement: Supplementary file 1 — File S1. IGSDPSP readiness questionnaire [file JIMD-43-279-s001.pdf]

James Lind Alliance
Priority Setting Partnerships

Prospective Priority Setting Partnerships: readiness questionnaire

The James Lind Alliance (JLA) works with Priority Setting Partnerships (PSPs) of patients and clinicians to identify uncertainties about treatments and healthcare interventions, and prioritise those for research. Organisations and individuals intending to form PSPs should be aware of the practical input needed to run a PSP, as well as the resources required. Details of the process are described in the JLA's Guidebook at www.jla.nihr.ac.uk .

Prospective PSPs are asked to complete the following questionnaire, to helpthem and the JLA ascertaintheir readiness to commence the priority setting process. Your answers will be for internal use by the JLA only, and will be treated as confidential. **If you are unsure about any areas, please contact us on jla@soton.ac.uk** .

Once you and the JLA agree that the PSP has everything in place to begin, you will be allocated an independent JLA adviser and you will contract directly with them. Please let the JLA adviser know if there are any issues that they need to be aware of with regard to contractual arrangements with your organisation (e.g. insurance requirements, payment terms, or any other expectations with regard to freelance workers or consultants).

We look forward to hearing from you.

Contact Details

**Name:**	Terry G. J. Derks
**Organisation:**	University of Groningen, UMC Groningen
**Email:**	t.g.j.derks@umcg.nl

**Proposed PSP topic area:**	Children and adults with hepatic Glycogen Storage Disease (GSD)

1. Please tell us about the **area of clinical or healthcare research** your PSP would address.

Glycogen storage diseases (GSD) are very rare inherited disorders of glycogen metabolism, the cumulative frequency of all types is approximately 1 in 20,000 to 43,000 live births. The different types of GSD have been categorized by number mostly in accordance with the chronological order, in which the protein defects were identified. The hepatic GSD types (and affected enzymes) are:

- type 0 glycogen synthase
- type I glucose 6-phosphatase (types Ia and Ib are further discriminated)
- type III debranching enzyme (types IIIa and IIIb are further discriminated)
- type IV branching enzyme
- type VI liverphosphorylase
- type IX liver phosphorylase kinase
- type XI glucose transporter-2

According to the textbooks, there are important differences between the hepatic GSD subtypes, including the age of onset, progression, other organ involvement, and clinical severity. But, as carbohydrate metabolism in the liver is responsible for

plasma glucose homeostasis, patients with hepatic GSD share many(presenting) symptoms and signs, including fasting intolerance, hepatomegaly, failure to thrive and hypoglycaemia. Dietary management and it's monitoring are the cornerstone in the follow-up for patients with hepatic GSD, regardless of the subtype.

2. It is important to consider the **scope** of the proposed PSP. Scope may be defined by the patient population of interest (eg, adults and or/children) or the breadth of the condition and the unique issues which sit within it. PSPs with broad scope are likely to receive more potential uncertainties. More resources will therefore be needed to process and check these, resulting in higher costs. Although the scope may not be finalised at this stage, what do you think the scope of the PSP will be? Have you considered how the scope of your PSP will influence how your priorities might be taken forward by research funders?

The scope of the proposed PSP is *children and adults with hepatic GSD*. Responses need to be categorized per:
- 'general' or 'specific GSD subtype';
- the geographic region (Europe, North-America, Latin- and South-America);
- age.

This scope may facilitate research projects for both general hepatic GSDs and specific subtypes.

3. The PSP leader has overall responsibility for successful delivery of the PSP. Who is responsible for **leading** this PSP, in terms of demonstrating commitment to the process, driving the PSP forward to completion and generating wider stakeholder engagement across the sector that the PSP will cover? Please explain their skills and networks to be able to complete this work. It is worth also considering at this time potential steering group members, particularly those with established networks of patients, carers and/or health professionals and credibility within these communities.

The PSP leader will be Terry Derks (MD, PhD), consultant in pediatric metabolic medicine, section of metabolic diseases, Beatrix Childrens' Hospital, UMC Groningen, University of Groningen. He is a principle investigator in the UMCG and coordinator of the UMCG GSD centre of expertise. Together with members of the scientific, patient and local organizing committees, he organizes the next International GSD Conference in Groningen, the Netherlands on 15-17 June 2017.

The PSP steering group covers:
- various health care professionals: physicians and a nurse. Ideally we will also invite 1-2 dietitians before or during IGSD2017.
- GSD patients who became health care professionals (NM, DC)
- health care professionals who have children with GSD (IF, ML, US)
- connection with professional networks like SSIEM, Metab-l, the dietitians listserver and MetabERN
- important direct patient network contacts with GSD patient associations in France (Association Francophone des Glycogénoses), Germany (Selbsthilfegruppe Glykogenose Deutchland e.V.), North-America (Association for Glycogen Storage Disease), Scandinavia (Scandinavian Association for Glycogen Storage Disease), Latin- and South-America (Glucolatino and Associação Brasileira de Glicogenose), Spain (Asociacion Española de Enfermos de Glucogenosis) and the Netherlands (Volwassenen Kinderen en Stofwisselingsziekten)
- connection with EURORDIS, a European non-governmental patient-driven alliance for rare diseases.

4. Who would be involved in the **day-to-day administration and co-ordination** of the PSP (eg organising teleconferences and meetings, writing action notes, managing communications with partners)? Depending on skills, other examples of work this person could also get involved with include preparing a website, running a Twitter feed for the PSP, and designing and communicating the survey. Do you have this support available?

Day-to-day administration and co-ordination of the PSP will be performed by the staff secretary of the section of metabolic diseases, Beatrix Childrens' Hospital, UMC Groningen, University of Groningen (Mrs Elma Daanje).

5. One of the most time-consuming and technical roles in a priority setting partnership is that of the information specialist, who will analyse the survey responses, search for relevant evidence, and formulate potential research questions. All of this forms a key quality assurance element of the PSP process. Please have a look at the relevant section of the JLA Guidebook. How will this work be undertaken? Have you identified an information specialist or someone else with the relevant skills to do this? Please describe any links you have to the Cochrane Collaboration.

The Information specialist will be Fabian Peeks, MD/PhD student at the section of metabolic diseases, Beatrix Childrens' Hospital, UMC Groningen, University of Groningen.
There are no links to the Cochrane Collaboration.

6. **Collaboration between patients/carers and clinicians** is an essential component of a PSP. How do you propose to collaborate and with whom?

Step 1: The following people have been invited for the PSP steering group:

- PSP lead:	**Terry Derks**, MD, PhD, consultant in pediatric metabolic medicine, section of metabolic diseases, Beatrix Childrens' Hospital, UMC Groningen, University of Groningen.
- JLA Adviser:	Not yet determined
- Information Specialist:	**Fabian Peeks**, MD/PhD student at the section of metabolic diseases, Beatrix Childrens' Hospital, UMC Groningen, University of Groningen
- PSP Project Co-ordinator:	**Elma Daanje**, staff secretary at the section of metabolic diseases, Beatrix Childrens' Hospital, UMC Groningen, University of Groningen
- PSP steering group members:	**Antal Nemeth**<br>**David Weinstein**<br>**Nerea López Maldonado**<br>**Anne Hugon**<br>**Marcus Landgren,** father to a child with GSD1b, MD and President of the Scandinavian Association for Glycogen Storage Disease<br>**Iris Ferecchia**<br>**Ute Stachelhaus-Theimer,** mother of a patient with GSD I a, MD, head of the liver GSD department of the German Association of Glycogen Storage Disease.<br>**Damian Cohen**<br>**Alberto Ferriani**

After formal agreement with JLA, the PSP steering group will actively reach out to members from the UK and Italy.

Step 2: An initial teleconference was organised on 13 Dec 2017, in which a first draft of the 'readiness questionnaire' was discussed.

Step 3: The final 'readiness questionnaire' was shared with JLA on 16 Dec 2017.

Net steps will follow the diagram of the PSP process, including three important dates:
- IGSD2017 in Groningen, the Netherlands, 15-17 June 2017:
    Initial awareness meeting (± 1 day, before or after the meeting)
    At the conference, as plenary session presentation by our JLA Adviser
- SAGSD conference in Angelholm, Sweden, 28-29 April 2018:
- SHG conference in Duderstadt, Germany, 6-8 April 2018
    These could serve as intermediate and/or final PSP workshops

- At this stage we miss an experienced dietitian. After formal agreement with JLA, the PSP steering group will actively reach out to two experienced dietitians (first options: Maike Grotzke-Leweling and Carina Heidenborg). Moreover, during IGSD2017 there will be a Meet the Expert session organized by a group of clinically experienced metabolic dietitians. We will ask one of these organizers to join our group.
- Options for scheduling the Initial awareness meeting during IGSD2017. The ideal scenario would be:

- plenary session presentation by the JLA Advisor;
- network session after the plenary presentation;
- PSP steering group meeting at Saturday afternoon.

7. The **costs of running a PSP** depend on the expertise and staffing resources which can be sourced from within the participating organisations. Attached you will find a spreadsheet which shows examples of potential costs, based on the experience of previous PSPs. This is only an example and costs of running a PSP can vary considerably. What funding/resources do you and your partners have available? In the box below, please provide estimates of the resources you have available.

Costs for the PSP lead, Information Specialist, PSP Project Co-ordinator (organization of teleconferences), the survey software (survey monkey) and extra EUR 5,000 will be covered by the UMCG. Cost may be high due to the international character of the PSP Steering Group. However, we agreed that (1) meetings will be organized around IGSD2017 and subsequent national GSD meetings, and (2) PSP steering group members take care of their own travel costs. We will study the possibilities for small additional financial support within the national GSD associations.

8. The **outputs** of the PSP are likely to be uncertainties that will be presented to research funders. Who do you think might be the potential funders of research resulting from the top 10?

Potential funders of research resulting from the top 10 will be (inter)national organizations, NGO’s, research institutions, (pharmaceutical and medical food) industry and patient organizations.

9. As well as funded research, what other **outcomes** would you like to see from the top 10? What is the audience for your results and how will you disseminate them?

Additional outcomes that we would like to see from the top 10:
- Presentation at (inter)national conferences.
- Open access scientific publication.
- Reimbursement of the costs for medical care is a great concern and is not generally covered for patients/parents by (national) health care programs. The top 10 list may define the minimal standard of care and thereby identify minimal requirements that should be covered by the national health care programs.
- Approach towards an International GSD Society, in which health care professionals, patient organization representatives collaborate, organize (bi-annual?) meetings and define the governance structure towards industrial partners.
- Proof-of-principle for (a) governance structure, (b) patient participation (c) at a global scale, that may stimulate discussions and processes within MetabERN.

10. What will be the **key challenges** to running a PSP in this particular topic area? How do you propose to address them?

We expect the following key challenges:

<u>1. Several international cross-border aspects:</u> (a) In most projects PSP Steering Group Members originated from one country (i.e. UK), but this PSP Steering group currently originates from eight countries; (b) Language differences, both between PSP Steering Group Members (50% is non-natively English speaking) and when gathering uncertainties through international surveys; (c) In terms of differences in health care priorities between countries.
=>Between the PSP Steering Group Members, English will be the language of communication.
=>Surveys will be translated in English, Dutch, German, French, Spanish and Portuguese.
=>Further analyses need to address differences between geographic areas.
<u>2. The extremely low prevalence of the disorders.</u>

=> This may reveal important information regarding minimal requirements for health care.
=>It may serve as a proof of principle of how to ensure certain governance towards industry and patient participation and empowerment in the area of (ultra-)rare diseases, and thereby for the MetabERN network.
=> For health care professionals, clinical care and research are intimately connected.
3. There are general hepatic GSD topics but there may also be GSD subtype specific topics that need to be covered.
=>Further analyses need to address differences between GSD subtypes. People answering the survey should clarify who they are (clinician, dietitian, nurse, patient, parent) and which GSD subtype they represent.
4. Selection bias if only GSD patient organization members would be included.
=> Dissemination of the survey should not only take place through patient organizations, but also social media (Facebook), metabolic physicians (Metab-l, national professional societies) and metabolic dietitians' listserver. Information about the process will also be shared on the IGSD2017-website and this conference information is shared through social media, too.
5. There has already been published a Top 10 list for diabetes mellitus type I (DMI). Given the existing similarities (in terms of monitoring glucose homeostasis and organization of health care) and differences (rarity, funding opportunities for research and reimbursement of basic health care for individual patients) between DMI and hepatic GSD, it will be very interesting to compare the outcomes between these two disorders.
6. In the area of ultra rare diseases, there is limited funding for research and organizing conferences. Generally, the pharmaceutical and medical food industries sponsor these meetings.

11. **Transparency** of interests is important to the JLA. Will your PSP have links to the pharmaceutical industry? Will it have particular political/policy aims? Please describe. How will you demonstrate openness, transparency and inclusivity in the PSP process?

There will be no direct link to pharmaceutical or medical food industry. 'Transparency of interests' is a better description than 'conflicts of interest', and covers more than we usually disclose. The PSP steering group members will declare their links to the industry and other (potential) conflicts of interest. As an example, hereby Terry Derks presents his transparency of interests. If additional statements are requested by JLA, we will provide them.

I have no direct potential conflicts of interests for this PSP project. However, my profession is naturally associated with positions, that may conflict, as I am:
- coordinator of 2 centers of expertise (for patients with hepatic GSD and fatty acid oxidation) that have been endorsed by the Dutch Minister of Health, since 2015, who needs to fulfil requirements for accreditation;
- physician following ± 160 patients with hepatic GSD;
- principle investigator at the UMCG. The current UMCG requirement for PI's is publication of at least 8 manuscripts in peer-reviewed Q1 scientific journals in 3 years. I supervise PhD students on this topic, publications are a requirement to obtain the degree. Financial support for open access publications is gained independently.
- teacher at the University of Groningen;
- steering group member of MetabERN and co-coordinator of the 'sugar and fat group';
- chairman of IGSD2017;
- member of the clinical advisory board for Dimension Therapeutics;
- member of the trial steering committee of the GLYDE-trial and institutional PI (sponsor: Vitaflo);

Besides institutional and NGO grants, in the last 5 years I have received:
- research fees from Sigma Tau and Vitaflo;
- speaker's fees from Danone Nutricia and Vitaflo;
- training support from Genzyme;
- travel reimbursements from several GSD patient organizations, Dimension Therapeutics, Vitaflo, SSIEM
- fees from companies, institutions and patient organizations to organize IGSD2017 (see www.igsd2017.com).

I have agreed confidentiality agreements with approximately 10 (pharmaceutical) companies. All agreements follow the UMCG institutional rules through the Contract Research Office and legal department and are primarily signed by the Board of Directors of the UMCG and co-signed by me. All payments are directed to the institution and used for research and teaching purposes of our group.

12. Do you have a **start date** in mind for the PSP? PSPs typically take between 12 – 18 months to complete and there is alead-in time required to set up a steering group before you can progress. Is there a key date or event in the topic area which will influence your launch?

The IGSD2017 meeting will be in Groningen, the Netherlands on 15-17 June 2017.This would be the best opportunity for the Initial awareness meeting and a plenary session presentation by our JLA Adviser. Thereafter, next meetings will be organized during conferences of national GSD associations.

13. Is there **anything else** which you would like us to know about your prospective PSP?

No.

Thank you. Please return by email to jla@soton.ac.uk

Details correct October 2015

Working in partnership with

NHS
National Institute for
Health Research
