# Supplementary material for: Research priorities for liver glycogen storage disease: An international priority setting partnership with the James Lind Alliance
Source: J Inherit Metab Dis. 2019 Nov 13;43(2):279–89. doi: 10.1002/jimd.12178 (PMC7079148; doi:10.1002/jimd.12178)
Supplement: Supplementary file 2 — File S2. First IGSDPSP survey for identification of uncertainties [file JIMD-43-279-s002.pdf]

IGSD PSP

Help set priorities for future liver Glycogen Storage Disease (GSD) research.

Do you or does a family member have liver GSD? Do you work with patients with liver GSD?

We need your help in guiding professionals to improve the management and care of all types of liver GSD. We will make your voice heard!

For more information, send an email to igsdpsp@gmail.com, visit our website, or visit the website from the *James Lind Alliance*.

Your questions

What questions or concerns do you have about the management and/or care of liver GSD?

Question 1:

Question 2:

Question 3:

Your information

* Tick the boxes that describe your role (multiple answers possible).

- [ ] A person with liver GSD
- [ ] A parent, caregiver or relative of someone with liver GSD
- [ ] A health care professional
- [ ] I do not want to share this information
- [ ] Other (please specify)

* I am a

- Doctor
- Nurse
- Dietitian
- I don't want to share this information
- Other type of health care professional (please specify)

For patients:

What is your type of liver GSD?

For parents/caregivers/relatives:

What is the patient's type of liver GSD?

For patients:

What is your age (in years)? Please fill this in in the first slot (patient 1).

For parents/caregivers/relatives:

How old is your child (in years)?/How old are your children (in years)?

Patient 1

Patient 2

Patient 3

Patient 4

For healthcare professionals:

What type of GSD patients do you take care of?

- ☐ GSD type 0
- ☐ GSD type Ia
- ☐ GSD type Ib
- ☐ GSD type III
- ☐ GSD type IV
- ☐ GSD type VI
- ☐ GSD type IX
- ☐ GSD type XI / Fanconi-Bickel syndrome
- ☐ Unclassified / Unknown
- ☐ I do not want to share this information
- ☐ Other (please specify)

For healthcare professionals:

How many years have you been involved in the care of persons with liver GSD?

- ◯ 0-5
- ◯ 6-10
- ◯ 11-15
- ◯ 16-20
- ◯ >20
- ◯ I do not want to share this information

For (parents/caregivers/relatives of) patients:

In which country do you live?

For healthcare professionals:

In which country do you work?

Your contact details

If you would like to be kept informed about the next stages of this project, please leave your email address. Your information will be stored securely and will not be used for any other purpose than communication about this project.

Thank you!

Thank you for participating in the International liver Glycogen Storage Disease Priority Setting Partnership Survey.

For more information, send an email to igsdpsp@gmail.com, visit our website, or visit the website from the *James Lind Alliance*.
