# Supplementary material for: Research priorities for liver glycogen storage disease: An international priority setting partnership with the James Lind Alliance
Source: J Inherit Metab Dis. 2019 Nov 13;43(2):279–89. doi: 10.1002/jimd.12178 (PMC7079148; doi:10.1002/jimd.12178)
Supplement: Supplementary file 3 — File S3. Second IGSDPSP prioritization survey [file JIMD-43-279-s003.pdf]

IGSD PSP

Help us again in setting research priorities for liver GSD!

From our first survey which closed in December 2017, we received1388 questions on on the care and/or management of liver GSD raised by 763 responders from 58 countries!
From these questions, the International liver Glycogen Storage Disease Priority Setting Partnership (IGSD PSP) has produced 72 summary questions. These were produced by summarizing similar questions and by excluding questions that were already answered.

About this survey
We need you again to tell us which of the summary questions are most important for research to answer!

We want to hear again from:
*** people with liver GSD**
*** parents/caregivers/relatives from people with liver GSD**
*** partners from people with liver GSD**
*** healthcare professionals**

You do not need to know about research – just tell us what is important to you based on your own experience. Your answers will be stored and analysed anonymously.

Up to 25 questions will be taken to a final workshop in May 2019 in Germany where the top 10 will be agreed upon by members of the GSD community that are not involved in research. With this top 10 we will raise awareness for liver Glycogen Storage Disease and approach funders to finance the research that is needed to answer these questions.

How this survey works
You will help prioritize these questions in four steps:
Step 1: You will first be asked to read the long list of questions and choose all questions that you are interested in.
Step 2: From all these questions, you can choose the top 10 questions that you want research to answer.
Step 3: You are asked a few general questions on your role in the GSD community.
Step 4: Finally you are able to give us your contact details if you want to stay updated on the process.

For more information, send an email to igsdpsp@gmail.com, visit our website, or visit the website from the James Lind Alliance.
For regular updates on the project, please follow us on twitter @igsdpsp or facebook.

Step 1: Selecting your priorities for reseach

Please look through this list of questions about care and/or management of liver Glycogen Storage Disease. The list is fairly long so it is important that you read through to the end. Each question has a short code at the end - please ignore this, it is for the project team's reference only

Choose all the questions that interest you and that you most want research to answer on the care and/or management of liver Glycogen Storage Disease. In the next step you are able to choose your top 10 questions. Please note that you are not being asked to answer the questions!

* 1. Which of the following questions do you most want research to answer for liver Glycogen Storage Diseases?

- [ ] **How can we better monitor metabolic control and outcomes at different stages of life in patients with Glycogen Storage Disease?** G1AO1
- [ ] **How is the (natural) progression of liver Glycogen Storage Disease at different stages of life?** G1Age1
- [ ] **How can all health care providers involved (including experts) contribute to shared care for individual patients with liver Glycogen Storage Disease?** G1CC1
- [ ] **What is the relationship between carriership of liver Glycogen Storage Disease and symptoms and signs?** G1Car1
- [ ] **How can we improve the diagnostic procedures of liver Glycogen Storage Disease?** G1DP1
- [ ] **How can we better understand differences in disease severity by investigating patients with liver Glycogen Storage Disease?** G1DS1
- [ ] **What are costs and effects of ongoing care for patients with liver Glycogen Storage Diseas and their families?** G1E1
- [ ] **How important is climate/weather for patients with liver Glycogen Storage Disease?** G1CW1
- [ ] **How can we improve genetic counseling and preconception care for patients and families with liver Glycogen Storage Disease?** G1Gen1
- [ ] **What is the worldwide frequency (prevalence and incidence) of liver Glycogen Storage Disease?** G1Inc1
- [ ] **How can we help (families of) patients with liver Glycogen Storage Disease to encourage patients' independency?** G1Ind1
- [ ] **What are the consequences of consumption of alcohol and drugs for patients with liver Glycogen Storage Disease?** G1AD1
- [ ] **Should care be differentiated between male and female patients (with the same mutations) with liver Glycogen Storage Disease?** G1MF1
- [ ] **What are the alarm symptoms of patients with liver Glycogen Storage Disease and how can they be recognized?** G1Mon2
- [ ] **What is the role for new methods for monitoring metabolic control (like noninvasive continuous glucose and lactate measurements, new biomarkers) for patients with liver Glycogen Storage Disease?** G1Mon3
- [ ] **How can we improve counselling and perinatal management for patients with liver Glycogen Storage Disease?** G1Pre1

- [ ] **Is (population) neonatal screening possible for liver Glycogen Storage Disease?** G1Scr1
- [ ] **How can we improve the quality of life of patients with liver Glycogen Storage Disease?** G1QoL1
- [ ] **Should there be more information and research on synergistic heterozygosity / mixed liver Glycogen Storage Disease?** G1SH1
- [ ] **How to prevent and/or treat muscle problems in patients with liver Glycogen Storage Disease?** G1Com1a
- [ ] **How to prevent and/or treat heart problems in patients with liver Glycogen Storage Disease?** G1Com1b
- [ ] **How to prevent and/or treat kidney problems in patients with liver Glycogen Storage Disease?** G1Com1c
- [ ] **How to better prevent and/or treat intestinal problems in patients with liver Glycogen Storage Disease?** G1Com1d
- [ ] **How to prevent and/or treat liver problems in patients with liver Glycogen Storage Disease?** G1Com1e
- [ ] **How to prevent and/or treat oncological problems (i.e. leukemia) in patients with liver Glycogen Storage Disease?** G1Com1f
- [ ] **How to prevent and/or treat immunological problems (i.e. infections) in patients with liver Glycogen Storage Disease?** G1Com1g
- [ ] **How to prevent and/or treat hormonal problems (i.e. thyroid, menstrual cycle, growth, diabetes, insulin response) in patients with liver Glycogen Storage Disease?** G1Com1h
- [ ] **How to prevent and/or treat neurological problems in patients with liver Glycogen Storage Disease?** G1Com1i
- [ ] **How to prevent and/or treat hematological problems (i.e. anemia) in patients with liver Glycogen Storage Disease?** G1Com1j
- [ ] **How to prevent and/or treat dental problems in patients with liver Glycogen Storage Disease?** G1Com1k
- [ ] **How to prevent and/or treat psychiatric problems (i.e. depression) in patients with liver Glycogen Storage Disease?** G1Com1l
- [ ] **How to prevent and/or treat hyperlipidemia and its complications in patients with liver Glycogen Storage Disease?** G1Com1m
- [ ] **What is the optimal management of reduced bone mineral density and its complications (i.e. osteoporosis) in patients with liver Glycogen Storage Disease?** G2Ost1
- [ ] **Can liver Glycogen Storage Disease cause developmental delays throughout childhood?** G2DD1
- [ ] **How can we optimize treatment to prevent growth delays in liver Glycogen Storage Disease?** G2GD1
- [ ] **What is the life expectancy of patients with liver Glycogen Storage Disease?** G2LE1
- [ ] **What is the mechanism behind neutropenia and Inflammatory Bowel Disease (IBD) in Glycogen Storage Disease and can these complications be cured?** G2IBD1
- [ ] **What is the best therapy for neutropenia and infections (i.e. G-CSF or alternatives considering outcomes, complications and side effects (i.e. bone pain) in patients with Glycogen Storage Disease Type Ib (or Ia)?** G2Neu1
- [ ] **What is the optimal therapy (Modulen or alternatives) for Inflammatory Bowel Disease (IBD) and acute flares in patients with Glycogen Storage Disease Type Ib?** G2IBD2
- [ ] **How do you prevent, monitor and manage liver adenomas in liver Glycogen Storage Disease?** G2Ade1
- [ ] **What are the predictors and diagnostics of malignant transformations of liver adenomas in patients with liver Glycogen Storage Disease?** G2Ade2

- [ ] **When should liver transplantation be considered in patients with liver Glycogen Storage Disease and what are the (dis)advantages and long-term outcomes?** G2LT1
- [ ] **How does liver Glycogen Storage Disease affect the cognitive development of patients?** G2CD1
- [ ] **Which strategies could be useful to motivate adult patients with liver Glycogen Storage Disease to adhere to treatment?** G2Com1
- [ ] **How does liver Glycogen Storage Disease affect patients and families psychologically?** G2Psy1
- [ ] **What is the need for supplementation of micronutrients (i.e. vitamins and calcium) in patients with liver Glycogen Storage Disease?** G3Sup1
- [ ] **Which is the role and use of medium-chain triglycerides (MCT) in the management of different patients with liver Glycogen Storage Disease?** G3MCT1
- [ ] **What are the effects of different kinds of Ketogenic Diet in patients with Glycogen Storage Disease Type III?** G3KD1
- [ ] **What is the needed restriction of lactose, fructose or saccharose in different types of liver Glycogen Storage Disease?** G3DR1
- [ ] **How to manage diet regimen in relation to "before, during and after" physical exercise (sport, playing) for patients with liver Glycogen Storage Disease?** G3PE1
- [ ] **What are the long-term complications (liver, renal, gut) of a diet rich in uncooked cornstarch and/or high protein and should the diet be adjusted to prevent complications in liver Glycogen Storage Disease?** G3DT1
- [ ] **Can guidelines be made for patients with liver Glycogen Storage Disease and their caregivers about how to deal with behavioral problems and management of GSD diet, such as social consequences, lack of appetite/eating refusal, motivation, and sleeping disorders?** G3DT2
- [ ] **What is the best way to start dietary treatment, finding the optimal doses, and to administer the diet for patients with liver Glycogen Storage Disease?** G3DT3
- [ ] **How can existing cornstarch preperations be modified or alternative treatments be implemented that are easier to administer and/or keep blood sugar levels more stable for patients with liver Glycogen Storage Disease?** G3DT4
- [ ] **How can we individualize the diet and the adjustment regarding macronutrients (fats, carbohydrates proteins) and micronutrients (i.e. vitamins and calcium) for patients with liver Glycogen Storage Disease?** G3DT5
- [ ] **How does dietary intake impact on metabolic control in patients with liver Glycogen Storage Disease?** G3MC1
- [ ] **What (laboratory) testing and with which frequency is optimal for monitoring patients with liver Glycogen Storage Disease?** G4Mon1
- [ ] **What are the target levels for metabolic testing in liver Glycogen Storage Disease?** G4Tar1
- [ ] **How should optimal metabolic control both clinically and biochemically (like lactate, ketones and/or lipids) be achieved in liver Glycogen Storage Disease?** G4Lab1
- [ ] **What can be done to prevent hypoglycemia or restore blood sugar to a safe level in patients with liver Glycogen Storage Disease?** G4Hypo1
- [ ] **What are the acute and chronic consequences of hypoglycemia in patients with liver Glycogen Storage Disease?** G4Hypo2
- [ ] **What is the role of continuous glucose monitoring in patients with liver Glycogen Storage Disease?** G4CGM1
- [ ] **How can the accuracy of glucose monitoring be improved to better control glucose and prevent hypoglycemia for patients with liver Glycogen Storage Disease?** G4GM1
- [ ] **How do body changes througout life impact blood sugars in patients with liver Glycogen Storage Disease?** G4LH1

☐ **How should sickness and emergency situations be managed for patients with liver Glycogen Storage Disease?** G4EM1

☐ **What are the best options (for example gene therapy or enzyme replacement therapy) for achieving sufficient amount of working enzyme in patients with liver Glycogen Storage Disease?** G4ERGT1

☐ **What are the risks and benefits of gene therapy for patients with liver Glycogen Storage Disease?** G4GTR1

☐ **Can consensus guidelines (for management) be achieved for patients with liver Glycogen Storage Disease?** G4Guide1

☐ **What are the side effects of over the counter drugs for patients with liver Glycogen Storage Disease?** G4OCD1

☐ **What are the risks and benefits of different options for overnight treatment for patients with liver Glycogen Storage Disease and how can we maximize safety?** G4Night1

☐ **How can we personalize treatment for patients with liver Glycogen Storage Disease?** G4Pers1

☐ **How can patients with liver Glycogen Storage Disease achieve and/or maintain a healthy weight throughout life?** G4Weight1

Step 2: Selecting your priorities for research

On the last page you have selected several questions. Of these questions, choose the 10 questions you most want research to answer for liver Glycogen Storage Disease.

* 2. Which 10 questions do you most want answered by research on the care and/or management of liver GSD?

- [ ] **How can we better monitor metabolic control and outcomes at different stages of life in patients with Glycogen Storage Disease?** G1AO1
- [ ] **How is the (natural) progression of liver Glycogen Storage Disease at different stages of life?** G1Age1
- [ ] **How can all health care providers involved (including experts) contribute to shared care for individual patients with liver Glycogen Storage Disease?** G1CC1
- [ ] **What is the relationship between carriership of liver Glycogen Storage Disease and symptoms and signs?** G1Car1
- [ ] **How can we improve the diagnostic procedures of liver Glycogen Storage Disease?** G1DP1
- [ ] **How can we better understand differences in disease severity by investigating patients with liver Glycogen Storage Disease?** G1DS1
- [ ] **What are costs and effects of ongoing care for patients with liver Glycogen Storage Diseas and their families?** G1E1
- [ ] **How important is climate/weather for patients with liver Glycogen Storage Disease?** G1CW1
- [ ] **How can we improve genetic counseling and preconception care for patients and families with liver Glycogen Storage Disease?** G1Gen1
- [ ] **What is the worldwide frequency (prevalence and incidence) of liver Glycogen Storage Disease?** G1Inc1
- [ ] **How can we help (families of) patients with liver Glycogen Storage Disease to encourage patients' independency?** G1Ind1
- [ ] **What are the consequences of consumption of alcohol and drugs for patients with liver Glycogen Storage Disease?** G1AD1
- [ ] **Should care be differentiated between male and female patients (with the same mutations) with liver Glycogen Storage Disease?** G1MF1
- [ ] **What are the alarm symptoms of patients with liver Glycogen Storage Disease and how can they be recognized?** G1Mon2
- [ ] **What is the role for new methods for monitoring metabolic control (like noninvasive continuous glucose and lactate measurements, new biomarkers) for patients with liver Glycogen Storage Disease?** G1Mon3
- [ ] **How can we improve counselling and perinatal management for patients with liver Glycogen Storage Disease?** G1Pre1
- [ ] **Is (population) neonatal screening possible for liver Glycogen Storage Disease?** G1Scr1
- [ ] **How can we improve the quality of life of patients with liver Glycogen Storage Disease?** G1QoL1
- [ ] **Should there be more information and research on synergistic heterozygosity / mixed liver Glycogen Storage Disease?** G1SH1
- [ ] **How to prevent and/or treat muscle problems in patients with liver Glycogen Storage Disease?** G1Com1a
- [ ] **How to prevent and/or treat heart problems in patients with liver Glycogen Storage Disease?** G1Com1b

- [ ] **How to prevent and/or treat kidney problems in patients with liver Glycogen Storage Disease?** G1Com1c
- [ ] **How to better prevent and/or treat intestinal problems in patients with liver Glycogen Storage Disease?** G1Com1d
- [ ] **How to prevent and/or treat liver problems in patients with liver Glycogen Storage Disease?** G1Com1e
- [ ] **How to prevent and/or treat oncological problems (i.e. leukemia) in patients with liver Glycogen Storage Disease?** G1Com1f
- [ ] **How to prevent and/or treat immunological problems (i.e. infections) in patients with liver Glycogen Storage Disease?** G1Com1g
- [ ] **How to prevent and/or treat hormonal problems (i.e. thyroid, menstrual cycle, growth, diabetes, insulin response) in patients with liver Glycogen Storage Disease?** G1Com1h
- [ ] **How to prevent and/or treat neurological problems in patients with liver Glycogen Storage Disease?** G1Com1i
- [ ] **How to prevent and/or treat hematological problems (i.e. anemia) in patients with liver Glycogen Storage Disease?** G1Com1j
- [ ] **How to prevent and/or treat dental problems in patients with liver Glycogen Storage Disease?** G1Com1k
- [ ] **How to prevent and/or treat psychiatric problems (i.e. depression) in patients with liver Glycogen Storage Disease?** G1Com1l
- [ ] **How to prevent and/or treat hyperlipidemia and its complications in patients with liver Glycogen Storage Disease?** G1Com1m
- [ ] **What is the optimal management of reduced bone mineral density and its complications (i.e. osteoporosis) in patients with liver Glycogen Storage Disease?** G2Ost1
- [ ] **Can liver Glycogen Storage Disease cause developmental delays throughout childhood?** G2DD1
- [ ] **How can we optimize treatment to prevent growth delays in liver Glycogen Storage Disease?** G2GD1
- [ ] **What is the life expectancy of patients with liver Glycogen Storage Disease?** G2LE1
- [ ] **What is the mechanism behind neutropenia and Inflammatory Bowel Disease (IBD) in Glycogen Storage Disease and can these complications be cured?** G2IBD1
- [ ] **What is the best therapy for neutropenia and infections (i.e. G-CSF or alternatives considering outcomes, complications and side effects (i.e. bone pain) in patients with Glycogen Storage Disease Type Ib (or Ia)?** G2Neu1
- [ ] **What is the optimal therapy (Modulen or alternatives) for Inflammatory Bowel Disease (IBD) and acute flares in patients with Glycogen Storage Disease Type Ib?** G2IBD2
- [ ] **How do you prevent, monitor and manage liver adenomas in liver Glycogen Storage Disease?** G2Ade1
- [ ] **What are the predictors and diagnostics of malignant transformations of liver adenomas in patients with liver Glycogen Storage Disease?** G2Ade2
- [ ] **When should liver transplantation be considered in patients with liver Glycogen Storage Disease and what are the (dis)advantages and long-term outcomes?** G2LT1
- [ ] **How does liver Glycogen Storage Disease affect the cognitive development of patients?** G2CD1
- [ ] **Which strategies could be useful to motivate adult patients with liver Glycogen Storage Disease to adhere to treatment?** G2Com1
- [ ] **How does liver Glycogen Storage Disease affect patients and families psychologically?** G2Psy1
- [ ] **What is the need for supplementation of micronutrients (i.e. vitamins and calcium) in patients with liver Glycogen Storage Disease?** G3Sup1

- [ ] **Which is the role and use of medium-chain triglycerides (MCT) in the management of different patients with liver Glycogen Storage Disease?** G3MCT1
- [ ] **What are the effects of different kinds of Ketogenic Diet in patients with Glycogen Storage Disease Type III?** G3KD1
- [ ] **What is the needed restriction of lactose, fructose or saccharose in different types of liver Glycogen Storage Disease?** G3DR1
- [ ] **How to manage diet regimen in relation to "before, during and after" physical exercise (sport, playing) for patients with liver Glycogen Storage Disease?** G3PE1
- [ ] **What are the long-term complications (liver, renal, gut) of a diet rich in uncooked cornstarch and/or high protein and should the diet be adjusted to prevent complications in liver Glycogen Storage Disease?** G3DT1
- [ ] **Can guidelines be made for patients with liver Glycogen Storage Disease and their caregivers about how to deal with behavioral problems and management of GSD diet, such as social consequences, lack of appetite/eating refusal, motivation, and sleeping disorders?** G3DT2
- [ ] **What is the best way to start dietary treatment, finding the optimal doses, and to administer the diet for patients with liver Glycogen Storage Disease?** G3DT3
- [ ] **How can existing cornstarch preperations be modified or alternative treatments be implemented that are easier to administer and/or keep blood sugar levels more stable for patients with liver Glycogen Storage Disease?** G3DT4
- [ ] **How can we individualize the diet and the adjustment regarding macronutrients (fats, carbohydrates proteins) and micronutrients (i.e. vitamins and calcium) for patients with liver Glycogen Storage Disease?** G3DT5
- [ ] **How does dietary intake impact on metabolic control in patients with liver Glycogen Storage Disease?** G3MC1
- [ ] **What (laboratory) testing and with which frequency is optimal for monitoring patients with liver Glycogen Storage Disease?** G4Mon1
- [ ] **What are the target levels for metabolic testing in liver Glycogen Storage Disease?** G4Tar1
- [ ] **How should optimal metabolic control both clinically and biochemically (like lactate, ketones and/or lipids) be achieved in liver Glycogen Storage Disease?** G4Lab1
- [ ] **What can be done to prevent hypoglycemia or restore blood sugar to a safe level in patients with liver Glycogen Storage Disease?** G4Hypo1
- [ ] **What are the acute and chronic consequences of hypoglycemia in patients with liver Glycogen Storage Disease?** G4Hypo2
- [ ] **What is the role of continuous glucose monitoring in patients with liver Glycogen Storage Disease?** G4CGM1
- [ ] **How can the accuracy of glucose monitoring be improved to better control glucose and prevent hypoglycemia for patients with liver Glycogen Storage Disease?** G4GM1
- [ ] **How do body changes througout life impact blood sugars in patients with liver Glycogen Storage Disease?** G4LH1
- [ ] **How should sickness and emergency situations be managed for patients with liver Glycogen Storage Disease?** G4EM1
- [ ] **What are the best options (for example gene therapy or enzyme replacement therapy) for achieving sufficient amount of working enzyme in patients with liver Glycogen Storage Disease?** G4ERGT1
- [ ] **What are the risks and benefits of gene therapy for patients with liver Glycogen Storage Disease?** G4GTR1
- [ ] **Can consensus guidelines (for management) be achieved for patients with liver Glycogen Storage Disease?** G4Guide1
- [ ] **What are the side effects of over the counter drugs for patients with liver Glycogen Storage Disease?** G4OCD1
- [ ] **What are the risks and benefits of different options for overnight treatment for patients with liver Glycogen Storage Disease and how can we maximize safety?** G4Night1

- [ ] **How can we personalize treatment for patients with liver Glycogen Storage Disease?** G4Pers1

- [ ] **How can patients with liver Glycogen Storage Disease achieve and/or maintain a healthy weight throughout life?** G4Weight1

Your information

Tick the box that best describes your role. We acknowledge that some people will fall into more than one group. For the survey it is important that you choose the aspect of your identity that feels most relevant to your answers

* 3. Tick the box that best describes your role

- ◯ A person with liver GSD
- ◯ A parent, caregiver, partner or relative of someone with liver GSD
- ◯ A health care professional
- ◯ I do not want to share this information

Step 3: Your information

* 4. I am a

- ○ Doctor
- ○ Nurse
- ○ Dietitian
- ○ I don't want to share this information
- ○ Other type of health care professional (please specify)

Step 3: Your information

5. **For patients:**
What is your type of liver GSD?
For parents/caregivers/relatives:
What is the patient's type of liver GSD?

6. **For patients:**
What is your age (in years)? Please fill this in in the first slot (patient 1).
For parents/caregivers/relatives:
How old is your child (in years)?/How old are your children (in years)?

Patient 1

Patient 2

Patient 3

Patient 4

7. **For healthcare professionals:**
What type of GSD patients do you take care of?

- ☐ GSD type 0
- ☐ GSD type Ia
- ☐ GSD type Ib
- ☐ GSD type III
- ☐ GSD type IV
- ☐ GSD type VI
- ☐ GSD type IX
- ☐ GSD type XI / Fanconi-Bickel syndrome
- ☐ Unclassified / Unknown
- ☐ I do not want to share this information
- ☐ Other (please specify)

8. **For healthcare professionals:**

How many years have you been involved in the care of persons with liver GSD?

- ◯ 0-5
- ◯ 6-10
- ◯ 11-15
- ◯ 16-20
- ◯ >20
- ◯ I do not want to share this information

Step 3: Your information

9. **For (parents/caregivers/relatives of) patients:**
In which country do you live?

10. **For healthcare professionals:**
In which country do you work?

Step 4: Your contact details

11. If you would like to be kept informed about the next stages of this project, please leave your email address. Your information will be stored securely and will not be used for any other purpose than communication about this project.

Thank you!

Thank you for participating in the International liver Glycogen Storage Disease Priority Setting Partnership Priority Setting Survey.

For more information, send an email to igsdpsp@gmail.com, visit our website, or visit the website from the *James Lind Alliance*.
For regular updates on the project, please follow us on twitter @igsdpsp or facebook.
