# Supplementary material for: Research priorities for liver glycogen storage disease: An international priority setting partnership with the James Lind Alliance
Source: J Inherit Metab Dis. 2019 Nov 13;43(2):279–89. doi: 10.1002/jimd.12178 (PMC7079148; doi:10.1002/jimd.12178)
Supplement: Supplementary file 5 — File S5. Original questions of summary question “How should sickness and emergency situations be managed for patients with liver Glycogen Storage Disease?” File S6. Total and group ranking of summary questions after the second prioritization survey. The top 10 and bottom 10 priorities are highlighted in green and red, respectively. *Rank after the second prioritization survey. HCP, Healthcare professional; J, joint rank [file JIMD-43-279-s005.docx]

**Supplementary File 5.** Original Questions of Summary Question “How should sickness and emergency situations be managed for patients with liver Glycogen Storage Disease?”

| Question ID | Original Question | Category | Survey | Responder | GSD type |
| --- | --- | --- | --- | --- | --- |
| 394,3 | What can be done about the emergency department of any hospital, with the intention for them to have enough knowledge with the protocol on a GSD? | Education/ treatment | English | Patient/Carer | Ia |
| 47,1 | It seems like many infants have issues with daily vomiting, multiple times a day. What can be done better to decrease or prevent this? It is very frustrating and draining to deal with GSD feeds every 90 min and then be dealing with cleaning up explosive vomit and re feeding all the time. | Treatment/ self-management | English | Carer | Ia |
| 57,1 | Is there a specific protocol to follow when a GSD patient gets sick? | Treatment | English | Healthcare professional | - |
| 268,1 | The care we receive in ER's | Treatment | English | Carer | Ia |
| 381,1 | Emergency protocols | Treatment | English | Patient/Healthcare professional | Ia |
| 437,1 | What is adequate treatment of patients with GSD who are acutely admitted clinically by general healthcare professionals? | Treatment | Dutch | Healthcare professional | - |
| 459,1 | Hypoglycemia hospital management | Treatment | German | Patient | Ia |
| 652,1 | How to treat the fever? Which medicine is the most indicated? | Treatment | Spanish | Carer | - |
| 661,1 | Emergency management | Treatment/ self-management | Spanish | Healthcare professional | - |
| 666,1 | How to act in an emergency | Self-management/ treatment | Spanish | - | VI |
| 668,1 | I worry to know that hospitals, where patients with hepatic GSD are being treated, do not have an emergency protocol established. We are always parents who have to give these guidelines | Treatment/ self-management | Spanish | Carer | Ia |
| 216,2 | Emergency situations, how to manage | Treatment | Spanish | Carer | 0 |
| 287,2 | Proper treatment at local ER's | Treatment | English | Carer | Ia |
| 475,2 | How to ensure that in the event of an emergency (incl. unconsciousness) it is ensured that the affected persons are recognized and treated correctly. | Self-management/ treatment | English | Patient | 0 |
| 620,2 | What to do in case of vomiting? | Treatment | German | Carer | Ia |
| 621,2 | How to act in front of a ketosis? | Treatment | Spanish | - | III |
| 626,2 | What to do in the case of an illness? with fever, vomiting ... | Treatment | Spanish | Patient/Carer | IX |
| 631,2 | Protocol when glucose and ketones are out of control | Treatment | Spanish | Carer | IX |
| 633,2 | In case of emergencies, if we do not have specialists | Treatment/ self-management | Spanish | Carer | IX |
| 678,2 | How to deal with emergency | Treatment/ self-management | Spanish | Healthcare professional | - |
| 51,3 | Sick diet | Treatment | English | Healthcare professional | - |
| 607,3 | Priority management in GSD emergencies | Treatment | Spanish | Carer | IX |
| 631,3 | Guidelines in case of illness | Treatment/ self-management | Spanish | Carer | IX |

**Supplementary File 6:** Total and group ranking of summary questions after the second prioritization survey.

| Summary Question | **Rank after Q2*** | Patient rank | Carer rank | HCP rank |
| --- | --- | --- | --- | --- |
| What are the long-term complications (liver, renal, gut) of a diet rich in uncooked cornstarch and/or high protein and should the diet be adjusted to prevent complications in liver Glycogen Storage Disease? | **1** | 9J | 1 | 3 |
| What are the risks and benefits of gene therapy for patients with liver Glycogen Storage Disease? | **2** | 1 | 2 | 12J |
| How can existing cornstarch preparations be modified or alternative treatments be implemented that are easier to administer and/or keep blood sugar levels more stable for patients with liver Glycogen Storage Disease? | **3** | 9J | 4 | 4 |
| What are the best options (for example gene therapy or enzyme replacement therapy) for achieving sufficient amount of working enzyme in patients with liver Glycogen Storage Disease? | **4** | 3J | 6 | 12J |
| How to manage diet regimen in relation to "before, during and after" physical exercise (sport, playing) for patients with liver Glycogen Storage Disease? | **5** | 5J | 3 | 14 |
| How should sickness and emergency situations be managed for patients with liver Glycogen Storage Disease? | **6J** | 9J | 7 | 18 |
| What is the needed restriction of lactose, fructose or saccharose in different types of liver Glycogen Storage Disease? | **6J** | 26J | 9 | 2 |
| How can patients with liver Glycogen Storage Disease achieve and/or maintain a healthy weight throughout life? | **8** | 3J | 8 | 33J |
| How can we individualize the diet and the adjustment regarding macronutrients (fats, carbohydrates proteins) and micronutrients (i.e. vitamins and calcium) for patients with liver Glycogen Storage Disease? | **9** | 13J | 18 | 16J |
| How to prevent and/or treat muscle problems in patients with liver Glycogen Storage Disease? | **10** | 2 | 24J | 22J |
| How is the (natural) progression of liver Glycogen Storage Disease at different stages of life? | **11** | 5J | 5 | 39J |
| How should optimal metabolic control both clinically and biochemically (like lactate, ketones and/or lipids) be achieved in liver Glycogen Storage Disease? | **12** | 20J | 32J | 5J |
| How do you prevent, monitor and manage liver adenomas in liver Glycogen Storage Disease? | **13** | 17J | 16J | 25J |
| How can we improve the quality of life of patients with liver Glycogen Storage Disease? | **14** | 13J | 10 | 39J |
| How can the accuracy of glucose monitoring be improved to better control glucose and prevent hypoglycemia for patients with liver Glycogen Storage Disease? | **15** | 26J | 12J | 30J |
| How to better prevent and/or treat intestinal problems in patients with liver Glycogen Storage Disease? | **16** | 20J | 11 | 39J |
| What is the role for new methods for monitoring metabolic control (like noninvasive continuous glucose and lactate measurements, new biomarkers) for patients with liver Glycogen Storage Disease? | **17J** | 40J | 24J | 8J |
| How to prevent and/or treat kidney problems in patients with liver Glycogen Storage Disease? | **17J** | 26J | 14J | 32 |
| What are the acute and chronic consequences of hypoglycemia in patients with liver Glycogen Storage Disease? | **19** | 19 | 12J | 43J |
| What are the effects of different kinds of Ketogenic Diet in patients with Glycogen Storage Disease Type III? | **20** | 20J | 48J | 7 |
| What are the risks and benefits of different options for overnight treatment for patients with liver Glycogen Storage Disease and how can we maximize safety? | **21** | 48J | 22J | 10J |
| What is the best way to start dietary treatment, finding the optimal doses, and to administer the diet for patients with liver Glycogen Storage Disease? | **22** | 34J | 37J | 10J |
| What are the consequences of consumption of alcohol and drugs for patients with liver Glycogen Storage Disease? | **23J** | 9J | 27 | 46 |
| How to prevent and/or treat hormonal problems (i.e. thyroid, menstrual cycle, growth, diabetes, insulin response) in patients with liver Glycogen Storage Disease? | **23J** | 5J | 32J | 45 |
| When should liver transplantation be considered in patients with liver Glycogen Storage Disease and what are the (dis)advantages and long-term outcomes? | **23J** | 48J | 29 | 5J |
| What are the alarm symptoms of patients with liver Glycogen Storage Disease and how can they be recognized? | **26J** | 17J | 19J | 47 |
| How does liver Glycogen Storage Disease affect the cognitive development of patients? | **26J** | 16 | 19J | 48J |
| What is the role of continuous glucose monitoring in patients with liver Glycogen Storage Disease? | **28** | 26J | 43J | 16J |
| How can we better monitor metabolic control and outcomes at different stages of life in patients with Glycogen Storage Disease? | **29J** | 40J | 30J | 19 |
| What (laboratory) testing and with which frequency is optimal for monitoring patients with liver Glycogen Storage Disease? | **29J** | 34J | 28 | 27J |
| What are the predictors and diagnostics of malignant transformations of liver adenomas in patients with liver Glycogen Storage Disease? | **31** | 26J | 43J | 21 |
| Can guidelines be made for patients with liver Glycogen Storage Disease and their caregivers about how to deal with behavioral problems and management of GSD diet, such as social consequences, lack of appetite/eating refusal, motivation, and sleeping disorders? | **32** | 45J | 14J | 33J |
| What is the need for supplementation of micronutrients (i.e. vitamins and calcium) in patients with liver Glycogen Storage Disease? | **33** | 34J | 32J | 30J |
| What can be done to prevent hypoglycemia or restore blood sugar to a safe level in patients with liver Glycogen Storage Disease? | **34** | 26J | 39J | 33J |
| What is the life expectancy of patients with liver Glycogen Storage Disease? | **35** | 26J | 22J | 52J |
| How to prevent and/or treat heart problems in patients with liver Glycogen Storage Disease? | **36J** | 13J | 41J | 48J |
| How does dietary intake impact on metabolic control in patients with liver Glycogen Storage Disease? | **36J** | 20J | 55 | 27J |
| How does liver Glycogen Storage Disease affect patients and families psychologically? | **38** | 26J | 16J | 63J |
| How can all health care providers involved (including experts) contribute to shared care for individual patients with liver Glycogen Storage Disease? | **39** | 20J | 30J | 57J |
| How to prevent and/or treat liver problems in patients with liver Glycogen Storage Disease? | **40** | 40J | 21 | 55J |
| What is the best therapy for neutropenia and infections (i.e. G-CSF or alternatives) considering outcomes, complications and side effects (i.e. bone pain) in patients with Glycogen Storage Disease Type Ib (or Ia)? | **41** | 54J | 43J | 20 |
| What is the optimal management of reduced bone mineral density and its complications (i.e. osteoporosis) in patients with liver Glycogen Storage Disease? | **42** | 40J | 56J | 22J |
| How do body changes throughout life impact blood sugars in patients with liver Glycogen Storage Disease? | **43** | 40J | 24J | 57J |
| What is the mechanism behind neutropenia and Inflammatory Bowel Disease (IBD) in Glycogen Storage Disease and can these complications be cured? | **44** | 48J | 47 | 27J |
| Which is the role and use of medium-chain triglycerides (MCT) in the management of different patients with liver Glycogen Storage Disease? | **45** | 54J | 68J | 1 |
| How can we optimize treatment to prevent growth delays in liver Glycogen Storage Disease? | **46** | 61J | 39J | 25J |
| How important is climate/weather for patients with liver Glycogen Storage Disease? | **47** | 20J | 41J | 71J |
| What is the relationship between carriership of liver Glycogen Storage Disease and symptoms and signs? | **48J** | 54J | 32J | 51 |
| Can consensus guidelines (for management) be achieved for patients with liver Glycogen Storage Disease? | **48J** | 71 | 58J | 8J |
| How to prevent and/or treat neurological problems in patients with liver Glycogen Storage Disease? | **50J** | 8 | 65 | 66J |
| What is the optimal therapy (Modulen or alternatives) for Inflammatory Bowel Disease (IBD) and acute flares in patients with Glycogen Storage Disease Type Ib? | **50J** | 54J | 48J | 37J |
| How can we personalize treatment for patients with liver Glycogen Storage Disease? | **50J** | 45J | 58J | 36 |
| How to prevent and/or treat immunological problems (i.e. infections) in patients with liver Glycogen Storage Disease? | **53** | 54J | 36 | 52J |
| How to prevent and/or treat psychiatric problems (i.e. depression) in patients with liver Glycogen Storage Disease? | **54** | 34J | 48J | 61J |
| How can we improve the diagnostic procedures of liver Glycogen Storage Disease? | **55J** | 48J | 54 | 43J |
| How to prevent and/or treat hyperlipidemia and its complications in patients with liver Glycogen Storage Disease? | **55J** | 68J | 62 | 15 |
| Can liver Glycogen Storage Disease cause developmental delays throughout childhood? | **57** | 45J | 43J | 61J |
| How can we better understand differences in disease severity by investigating patients with liver Glycogen Storage Disease? | **58J** | 34J | 61 | 55J |
| How can we help (families of) patients with liver Glycogen Storage Disease to encourage patients' independency? | **58J** | 61J | 37J | 52J |
| What are the target levels for metabolic testing in liver Glycogen Storage Disease? | **58J** | 61J | 67 | 22J |
| How to prevent and/or treat dental problems in patients with liver Glycogen Storage Disease? | **61** | 34J | 48J | 71J |
| Is (population) neonatal screening possible for liver Glycogen Storage Disease? | **62** | 68J | 53 | 37J |
| Which strategies could be useful to motivate adult patients with liver Glycogen Storage Disease to adhere to treatment? | **63** | 48J | 63J | 48J |
| How to prevent and/or treat oncological problems (i.e. leukemia) in patients with liver Glycogen Storage Disease? | **64** | 48J | 56J | 63J |
| What are costs and effects of ongoing care for patients with liver Glycogen Storage Disease and their families? | **65** | 54J | 48J | 66J |
| Should there be more information and research on synergistic heterozygosity / mixed liver Glycogen Storage Disease? | **66** | 65J | 66 | 42 |
| What are the side effects of over the counter drugs for patients with liver Glycogen Storage Disease? | **67** | 54J | 58J | 66J |
| How can we improve genetic counseling and preconception care for patients and families with liver Glycogen Storage Disease? | **68** | 68J | 63J | 57J |
| What is the worldwide frequency (prevalence and incidence) of liver Glycogen Storage Disease? | **69J** | 65J | 70 | 57J |
| How to prevent and/or treat hematological problems (i.e. anemia) in patients with liver Glycogen Storage Disease? | **69J** | 61J | 68J | 63J |
| How can we improve counselling and perinatal management for patients with liver Glycogen Storage Disease? | **71** | 65J | 72 | 66J |
| Should care be differentiated between male and female patients (with the same mutations) with liver Glycogen Storage Disease? | **72** | 72 | 71 | 66J |
